# Perfusion in Pedicled Skin Flaps: Initial Insights from Smartphone-Based Thermal Imaging Protocol

**DOI:** 10.3390/jpm14070730

**Published:** 2024-07-05

**Authors:** Lukas S. Fiedler, Burkard M. Lippert, Lukas Adrian, Tobias Meyer

**Affiliations:** 1 Department for Otorhinolaryngology/Head and Neck, Plastic Surgery, SLK Kliniken Heilbronn, 74078 Heilbronn, Germany; 2 Faculty of Medicine, Heidelberg University, 69120 Heidelberg, Germany; 3 Department of Otorhinolaryngology, Head and Neck Surgery, University of Tübingen, 72076 Tübingen, Germany

**Keywords:** skin flaps, smartphone diagnostics, thermal imaging, perfusion, pedicled flaps

## Abstract

Objective: Successful outcomes in head and neck surgery rely on maintaining perfusion in pedicled skin flaps. Thermal imaging offers a noninvasive means to assess tissue perfusion, potentially aiding in predicting flap viability. This pilot study explores the utility of SBTI (smartphone-based thermal imaging) for predicting flap vitality and monitoring during surgery. Methods: Thermal imaging was employed using the FLIR One System. An imaging protocol was established, defining points of interest (T1-T4) on pedicled skin flaps. Conducted over four months, the study integrated SBTI into reconstructive surgery for the face, head and neck defects post-tumor resections. SBTI’s effectiveness was assessed with n = 11 pedicled flaps, capturing images at key stages and correlating them with clinical flap assessment. Thermal images were retrospectively graded by two surgeons, evaluating flap perfusion on a scale from 1 to 5, based on temperature differences (1 = ΔT < 2 °C, 2 = ΔT ≥ 2 °C, 3 = ΔT ≥ 4 °C, 4 = ΔT ≥ 6 °C, and 5 = ΔT ≥ 8 °C), with assessments averaged for consensus and compared with the clinical assessment control group. Results: The study encountered challenges during implementation, leading to the exclusion of six patients. Patient data included 11 cases with n = 44 SBTI images. Intraoperative assessments consistently showed good perfusion. One postoperative dehiscence was noted, which retrospectively coincided with intraoperative SBTI grading, but not with clinical assessment. Statistical analysis indicated consistent outcomes following clinical and SBTI assessments. Thermal imaging accurately predicted flap viability, although it had limitations with small flaps. Conclusion: SBTI proved effective, inexpensive, and noninvasive for assessing tissue perfusion, showing promise for predicting flap viability and intraoperative monitoring in head and neck surgery.

## 1. Introduction

Skin defects in the head, face and neck resulting from chronic wounds, traumatic injuries, or oncological conditions require effective closure. A tailored approach must be developed for each defect based on the patient’s requirements and the extent of the affected tissues [1]. The reconstructive ladder is a tool used in plastic surgery to evaluate the extent of a defect and select the most appropriate method for treatment. This ladder begins with primary closure, followed by free skin grafts, local flaps, and pedicled flaps, and ends with free flaps, depending on the location and severity of the defect [2,3]. When reconstructing defects in the face, head, and neck, surgeons often use regional or distant pedicled flaps. Regional flaps are a suitable option for treating areas where the damage is not too severe and where it is crucial to maintain the natural look of the face. An understanding of the biomechanics of soft tissue and the vascular supply to skin flaps is elementary for assessing skin-/and flap perfusion [4].

Despite significant advancements in medical, surgical and technical fields, no technique has been found that allows for a consistent, simple, reliable, and cost-effective assessment of perfusion in cutaneous flaps. Therefore, direct clinical evaluation remains the gold standard in the monitoring of skin flaps [5,6]. Most head and neck surgeons evaluate flap perfusion based on parameters such as color, reperfusion, scratch testing (quality of the blood), and flap surface temperature [6,7].

Thermal imaging (TI), smartphone-based TI (SBTI), and dynamic infrared thermography (DIRT) have now shown promise as an innovative tool in several medical specialties. Using the detection of infrared radiation emitted by the body, thermography produces accurate visual representations of surface temperatures, allowing for areas of increased or decreased blood flow to be highlighted [8].

As an advanced, non-invasive, and low-cost, mostly smartphone-based (Teledyne FLIR LLC, Wilsonville, OR, USA) [9,10,11,12,13] imaging technique, SBTI offers valuable insights into the graduation of burn depths [9,10], as well as thermal physiology [14].

This study aimed to deliver an implementable algorithm for the use of SBTI in the perioperative setting and a prior insight into the accuracy of this TI technique as a reliable assessment for cutaneous perfusion in pedicled flaps in the face, head and neck.

## 2. Materials and Methods

### 2.1. Ethics Statement

This study involving human participants adheres to ethical standards. Approval was obtained from the Ethics Committee of the University of Heidelberg (Votum: S-630/2023) on 6 November 2023. The research conforms to the Declaration of Helsinki.

The publication of images and individual participant information is contingent on the authors securing free prior informed consent. The authors confirmed consent during the author’s license to publish without submitting the actual form. A standard patient consent form from Thieme© was obtained by all patients.

### 2.2. Thermal Imaging Technique

TI and dynamic infrared thermography (DIRT), have now shown promise as an innovative tool in several medical specialties [14]. Thermal imaging technology utilizes electromagnetic radiation in the near-infrared (NIR) range, specifically wavelengths ranging from 780 to 1400 nm. Unlike visible light, which is perceived by the human eye, NIR radiation is invisible and requires technology-based recording, analysis, and interpretation. By employing specialized cameras sensitive to NIR wavelengths, such as infrared cameras (e.g., the FLIR One System), it becomes possible to capture and visualize NIR radiation [15]. Using the detection of infrared radiation emitted by the body, thermography produces accurate visual representations of surface temperatures, allowing for areas of increased or decreased blood flow to be highlighted. Over these color-coded heat emission differences, indirect perfusion patterns can be drawn.

### 2.3. Implementation of SBTI in the Operating Room

**Figure 1:**
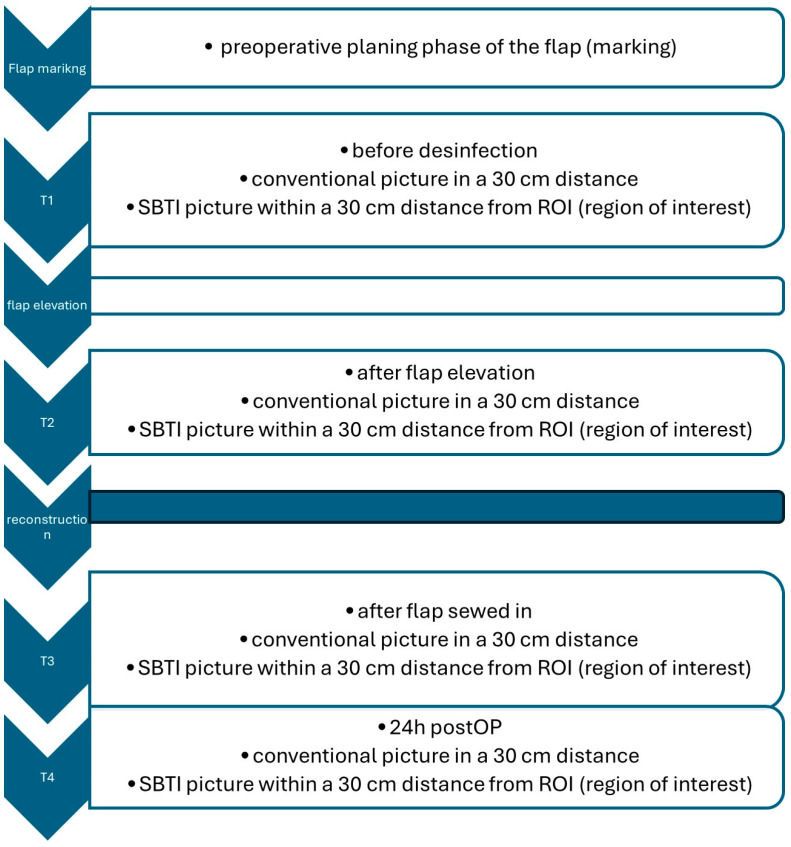
SBTI algorithm.

**Figure 2:**
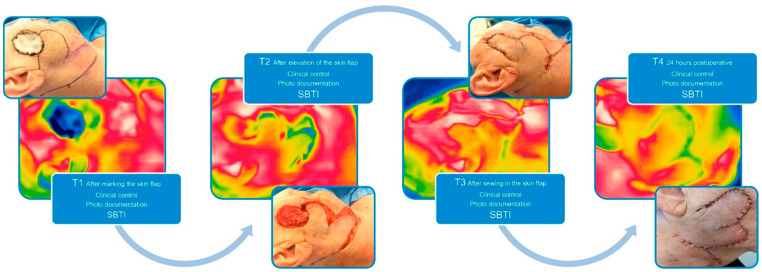
Implemented SBTI algorithm T1–4, showing the intraoperative picture in correlation to the SBTI picture.

Before the evaluation of the SBTI technique in cutaneous flaps, we established an imaging protocol, given in Figure 1; Figure 2, where we defined the definite points of interest (T1–T4). The ROI (region of interest) was defined by the center of the flap with an imaging distance taken 30 cm away from the central point of the flap. The room temperature in our OR is controlled by a thermoregulation system, which holds it constantly around 23 °C. The humidity of the room is constantly around 30%.

### 2.4. Study Course

Our study aimed to integrate thermal imaging into the assessment of patients undergoing reconstructive surgery for defects in the face and neck following tumor resections of varying extents. This investigation was conducted at SLK Kliniken Heilbronn, Germany, within the Department for Otorhinolaryngology/Head and Neck, Plastic Surgery, over four months, from November 2023 to March 2024. We integrated various data such as age, gender, diagnosis, disease stage, diabetes mellitus status, smoking history (pack years), hypertension, anticoagulants, history of previous surgeries in the operative area, details of the surgical procedure (including technique and date), location and size of the defect, intra-operative clinical assessment and Doppler signal, imaging details, postoperative complications, exact timing of post-operative assessments (T4) and the need for revision surgery followed up for two months.

Drawing from findings in prior research, we found that incorporating the FLIR One camera into our surgical protocol was straightforward, albeit requiring a learning curve for both its application and the interpretation of images about the indirect visualization of blood flow based on temperature differentials. Hence, we opted for the FLIR One System (Teledyne FLIR LLC, Wilsonville, OR, USA) for this pilot study. This multispectral camera features a near-infrared camera unit with a resolution of 160 × 120 pixels, covering a temperature range of 0 °C to 400 °C, with a thermal resolution of 70 mK.

For our investigation, we took thermal images from a total of n = 11 axial pattern and random pattern pedicled skin flaps of the face and neck (e.g., bilobed flaps, Rieger flaps, Hatchet flaps, island flaps, etc.) using the FLIR One camera, resulting in a total of n = 44 images. These images were captured at various stages: after marking (T1), after flap elevation (T2), upon completion of surgery (T3), and 24 h postoperatively (T4) (see Figure 2).

### 2.5. Scoring System

**Figure 3:**
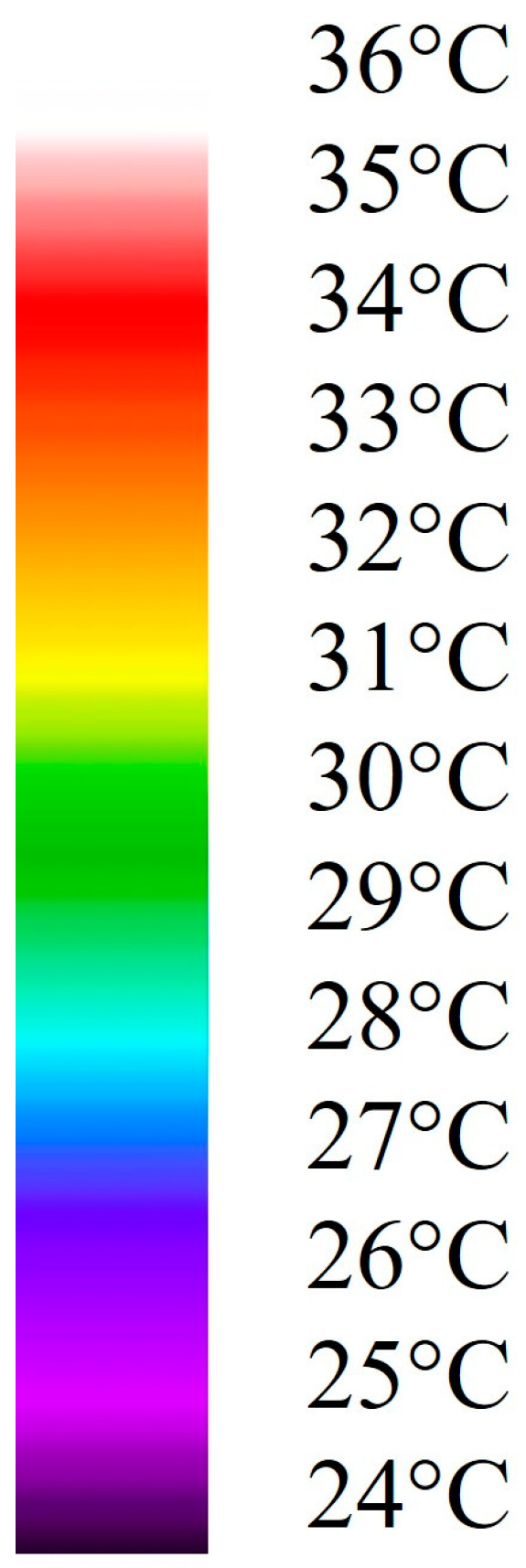
Rainbow temperature scale.

The SBTI study group was graded by 1 corresponding to ΔT = 0 °C, indicating perfect perfusion of the entire flap. Grades of 2, 3, 4, and 5 corresponded to ΔT ≥ 2 °C, ΔT ≥ 4 °C, ΔT ≥ 6 °C, and ΔT ≥ 8 °C, respectively, with grade 5 indicating no perfusion, from the flap center to the healthy periphery. For temperature interpretation, the rainbow temperature scale scheme was used (see Figure 3).

A control group was included, with clinical assessments used as the gold standard for comparison. The control group was graded on a scale from 1 to 5 based on the following criteria: a grade of 1 indicated good recapillarization (<1 s), no color difference between the flap and surrounding tissue, and peripheral flap microbleeding from its edges. A grade of 2 indicated good recapillarization (<2 s), no or low color difference, and peripheral flap microbleeding. A grade of 3 indicated recapillarization (>2 s), brighter flap color, and peripheral bleeding. A grade of 4 indicated no peripheral bleeding, delayed recapillarization signs, and a white flap. A grade of 5 indicated no peripheral bleeding, a pale flap, and no peripheral bleeding of the flap.

## 3. Results

### 3.1. Implementation

The implementation process for the study began with planning and drafting the study protocol inspired by Nischwitz et al. [1], without a cooling phase between the imaging points. (see Figure 3) Following this, the necessary SBTI camera was procured, and the implementation commenced. However, several obstacles were encountered during this phase. Initially, images were collected by different individuals (nursing staff) without adhering to the study protocol guidelines consequently. As a result, not all collected images could be used for the study and had to be excluded. Specifically, six patients were excluded due to the following reasons: inadequate distance (n = 2) or incomplete capture of the region of interest (n = 1). Additionally, patients were excluded if all required images from T1 to T4 were not available (n = 3), leading to a loss of follow-up. To address these issues, a team of two surgeons was involved in conducting SBTI in the operating room.

### 3.2. Patient Specifications

**Figure 4:**
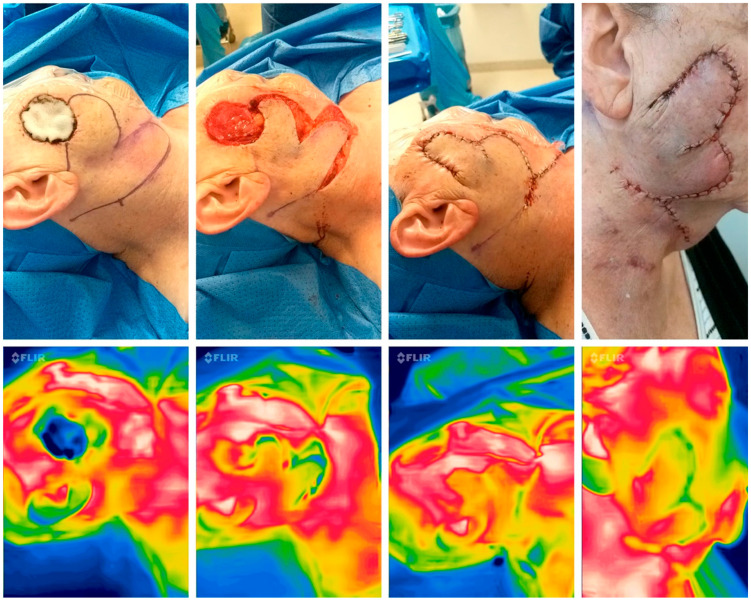
Perioperative thermal imaging in a random pattern flap with the FLIR one Pro Camera preoperatively (**left**), after flap elevation, after reconstruction, and 24 h after surgery (**right**) showing good perfusion due to the red color and minor yellow area in the periphery, which shows a low delta between normal and flap temperature.

**Figure 5:**
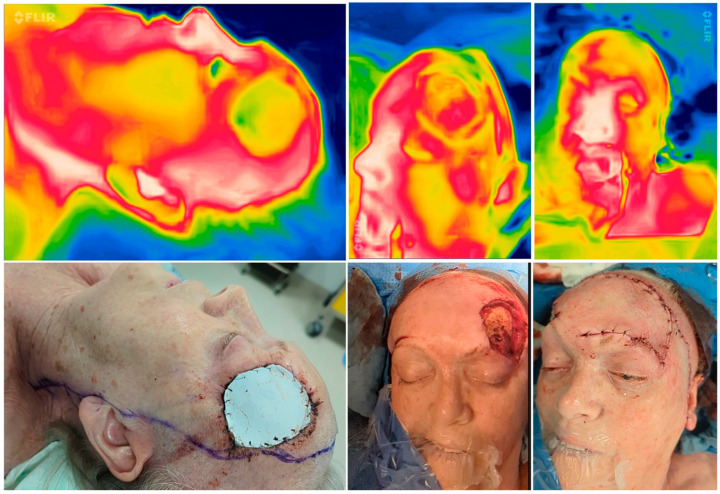
Perioperative thermal imaging in a combined reconstruction preoperatively (**left**), after flap elevation (**middle**), and after reconstruction (**right**) showing good perfusion due to a homogenous yellowish color emitted by the flaps (modified Hatchet and Esser flap).

The dataset encompasses a total of 11 (4 m; 7 f) patients of varying ages [64:93;77,37 yrs] who underwent reconstructive surgery for facial, head and neck defects [1 × 1 cm:6 × 6 cm] following diagnoses such as squamous cell carcinoma (SCC, n = 3), basal cell carcinoma (BCC, n = 6), malignant melanoma (MM, n = 1), or actinic keratosis (n = 1). Considering perioperative risk profiles, three patients had diabetes mellitus and six patients had hypertension. A total of two were smokers, with 30 and 40 pack years, respectively. The surgical techniques employed were diverse, including procedures such as Rieger flaps (n = 5), Esser flap (n = 1), bilobed flaps (n = 1, See Figure 4), two-island flaps, one transposition flap, and one Hatchet flap. In total, we included n = 3 axial pattern and n = 8 random pattern flaps. Surgery dates spanned from August 2023 to February 2024. Intraoperative assessments consistently indicated good perfusion, with a Doppler signal present in n = 1 cases, a Hatchet flap (Supratrochlear Artery, see Figure 5). One post-operative dehiscence in a cervical advancement flap was recorded which was treated by secondary intention, with revision surgery not occasionally necessary. Furthermore, this patient had an elevated complication risk due to his advanced age of 84. The intraoperative SBTI grading was “3” in T2 and T3 not in line with a grade of “2” at timepoints T2 and T3 in clinical assessment.

### 3.3. Descriptive Analysis of SBTI

As depicted in the two subsequent tables, the assessment of imaging data by both surgeons demonstrates consistent outcomes, albeit exhibiting a greater variability from time points T2–T4 in SBTI imaging (see Table 1 and Table 2).

### 3.4. Outcome

The pedicled skin flaps examined all showed a preserved, uniform thermal image signature peri- and postoperatively for time points T1–4 in both surgeons’ evaluations. This corresponded to a good postoperative result regarding the vitality of the tissue transfer in the clinical assessments 24 h and one week postoperatively. A single postoperative dehiscence occurred in a cervical advancement flap, which exhibited a diminished thermal signature in the periphery intraoperatively. Both evaluating surgeons consistently graded it as “3”, which means >4 °C difference from T2 to T4 in SBTI evaluations, while clinical assessments yielded a “2,” indicating a more favorable outcome. The dehiscence was not associated with diabetes or smoking.

### 3.5. Limitations

While the application of smartphone-based thermal imaging (SBTI) in flap monitoring presents several advantages, notable limitations must be considered. Thermal imaging demonstrated limited accuracy in small flaps approximately one centimeter in length, often exceeding the resolution capabilities of current technology and leading to potential misidentification. The practical use of SBTI is further constrained by its sensitivity to surface temperature changes, which can be influenced by external conditions such as room temperature differences, high humidity, and the use of cold wet gauze on the tissue. Additionally, anatomical challenges in regions like the nose and ears, and the impact of comorbidities such as smoking and diabetes mellitus, can skew thermal readings and reduce the reliability of SBTI in detecting true perfusion deficits.

For the depiction of our data, descriptive statistics were used. Inferential statistics are less useful for small sample sizes like n = 11 because the ability to generalize findings to a larger population is severely limited due to high variability and the increased risk of errors. Therefore, using descriptive statistics provides a clear and accurate summary of the data without making unsupported generalizations.

## 4. Discussion

Despite progressive medical and surgical, as well as technical developments, no technique has been found that permits a uniform, simple valid, and cost-effective postoperative flap assessment. As a result, direct clinical assessment remains the gold standard [5,6]. The use of thermal imaging to assess perfusion is not a new concept, in the identification of perforators in free and pedicled perforator flaps [16,17] and perfusion monitoring in free and pedicled flaps [13]. Multiple studies focus on the assessment of DIEP flaps [1] and lower limb reconstruction with propeller flaps [16] but, to our knowledge, no study was yet conducted using SBTI in head-/face-/and neck pedicled skin flaps. Further, we missed focus on implementation protocols and their obstacles in other studies.

In our study, we aimed to establish an implementable algorithm for SBTI use in the perioperative setting and evaluate its interpretability and accuracy in assessing cutaneous perfusion in pedicled flaps. The implementation of our SBTI protocol encountered initial challenges, including inconsistent image collection and exclusion of patients due to protocol deviations. Due to a late reaction on this topic, we had to exclude six patients, lowering our potential validity. However, with the involvement of a dedicated surgical team, these challenges were addressed, highlighting the importance of standardized protocols and interdisciplinary collaboration in adopting new technologies.

Our patient cohort consisted of individuals undergoing reconstructive surgery for various diagnoses, with diverse surgical techniques employed. Despite this heterogeneity, intraoperative assessments consistently indicated good perfusion, supporting the efficacy of our SBTI protocol in evaluating flap viability. Statistical analysis revealed consistent outcomes in both clinical and SBTI assessments, with SBTI demonstrating a greater variability at certain time points. Furthermore, the SBTI had a higher sensitivity due to our advancement flap which showed postoperative dehiscence. The dT was higher than 4 °C between the ROI in the center compared with the periphery of the flap, whereas the clinical assessment graded it by 2, not assuming a compromised perfusion state. Additionally, pedicled skin flaps showed preserved thermal signatures peri- and postoperatively, correlating with positive clinical outcomes and no further perfusion related complications.

The defined acquisition (T1–T4) of a thermal image signature as a surrogate marker for preserved tissue perfusion by SBTI proved to be simple, inexpensive, noninvasive, and efficient in the present sentinel study. The most relevant time points in SBTI seemed to be T2 and T3 in SBTI due to prognosis of perfusion complications in the postoperative frame, this should be further addressed in studies. Thermal imaging stands out among the assessment techniques due to its non-invasive nature and affordability, particularly when implemented via smartphone-based devices. It offers the advantage of intraoperative detection of vascular insults and provides valuable postoperative monitoring in pedicled flaps. However, there are some limitations of thermal imaging which can limit the accuracy. First, its utility is constrained by its limitation to surface temperature changes, and its specificity may vary depending on the circumstances. For example, using a cold wet gauze lying on the flap for several minutes, it normally takes 2–3 min until the superficial temperature in the TI resolves to its primary temperature. Depending on body composition, e.g., high subcutaneous fat proportion, which can decelerate rewarming of the tissue. Although cooling the tissue temperature may be of argument within perforator identification [1], it is not useful for the perfusion assessment in pedicled skin flaps via SBTI. Second, the evaluation of flaps of the nose and the ears showed continuous cold areas; therefore, an assessment with thermal imaging can bring inaccurate results. As a third point, smoking and diabetes mellitus can have a negative impact in the assessment of skin flaps due to perfusion impairment. These three factors of influence in thermal imaging could not be precisely addressed within our study and should be the focus of further studies.

In conclusion, while this pilot study demonstrates promising potential for assessing pedicled skin flap perfusion using smartphone-based thermal imaging (SBTI), several limitations warrant cautious interpretation of the results. The study’s small sample size and limited validation against established clinical methods reduce the generalizability of the findings, highlighting the need for larger, more rigorous studies. The accuracy of SBTI was limited in small flaps and anatomically challenging regions, such as the nose and ears, and was influenced by external factors like surface temperature changes and patient comorbidities, such as smoking and diabetes mellitus. To enhance the reliability and clinical utility of SBTI, future research should focus on increasing sample sizes, strengthening validation protocols, developing advanced imaging techniques, and refining assessment time points. Addressing these limitations will help build on the promising results of this pilot study and improve the efficacy of SBTI in clinical practice.

## 5. Conclusions

The concordance of the obtained thermal image signature with a positive clinical outcome suggests that the presented method is suitable for perioperative prognostic statements on postoperative flap vitality in pedicled skin flaps and for monitoring those during surgery. With the focus on time points T2 and T3 in SBTI if capable of being a prognosis factor due to wound healing and flap survival in the postoperative state, future studies should be conducted. The SBTI visualization method is an indirect indicator for perfusion and can be used for residents in training to establish a learning curve, reduce reaction time in flap failure and, as a consequence, maintain quality in surgical care. Due to the visualization of temperature differences, the method seems to work better in larger flaps than smaller ones. Further studies are necessary for the implementation of this method in clinical practice to support the surgeon’s intraoperative decision-making in the future.

## Figures and Tables

**Table 1:** Descriptive data from Surgeon No. 1 showing the clinical and SBTI assessments in T1–T4.

	Clinical Assessment T1	Clinical Assessment T2	Clinical Assessment T3	Clinical Assessment T4	SBTI Perfusion T1	SBTI Perfusion T2	SBTI Perfusion T3	SBTI Perfusion T4
Valid	11	11	11	11	11	11	11	11
Median	1.000	1.000	1.000	1.000	1.000	1.000	1.000	1.000
Mean	1.000	1.182	1.273	1.273	1.000	1.727	1.727	1.273
Std. Deviation	0.000	0.405	0.467	0.647	0.000	0.905	0.905	0.647
Variance	0.000	0.164	0.218	0.418	0.000	0.818	0.818	0.418
Minimum	1.000	1.000	1.000	1.000	1.000	1.000	1.000	1.000
Maximum	1.000	2.000	2.000	3.000	1.000	3.000	3.000	3.000

**Table 2:** Descriptive data from Surgeon No. 2 showing the clinical and SBTI assessments in T1–T4.

	Clinical Assessment T1_1	Clinical Assessment T2_1	Clinical Assessment T3_1	Clinical Assessment T4_1	SBTI Perfusion T1_1	SBTI Perfusion T2_1	SBTI Perfusion T3_1	SBTI Perfusion T4_1
Valid	11	11	11	11	11	11	11	11
Median	1.000	1.000	1.000	1.000	1.000	1.000	1.000	1.000
Mean	1.000	1.091	1.273	1.182	1.000	1.545	1.455	1.273
Std. Deviation	0.000	0.302	0.467	0.405	0.000	0.688	0.688	0.647
Variance	0.000	0.091	0.218	0.164	0.000	0.473	0.473	0.418
Minimum	1.000	1.000	1.000	1.000	1.000	1.000	1.000	1.000
Maximum	1.000	2.000	2.000	2.000	1.000	3.000	3.000	3.000

## Data Availability

Data is available on request.

## Previous Publication

The contents of this paper were presented under the title “SBTI for evaluation of regional skin flaps in the face, head and neck” at the “Crossing Borders” 95. German annual ENT Congress organized by the German Society of Otorhinolaryngology, Head and Neck Surgery in Essen, Germany on 15 May by Lukas Adrian.
